# Management of Hypertension in CAKUT: Protective Factor for CKD

**DOI:** 10.3389/fped.2019.00222

**Published:** 2019-06-04

**Authors:** Marina M. Gabriele, Paulo C. Koch Nogueira

**Affiliations:** ^1^Pediatric Nephrology Department, Instituto da Criança Hospital das Clínicas, University of São Paulo Medical School, São Paulo, Brazil; ^2^Pediatric Nephrology Department, UNIFESP–Escola Paulista de Medicina and Samaritano Hospital of São Paulo, São Paulo, Brazil

**Keywords:** CAKUT, chronic kidney disease (CKD), hypertension, renal disease progression, risk factor, children, blood pressure

## Abstract

Patients with congenital kidney and urinary tract abnormalities (CAKUT) will often develop end-stage renal disease at some point and the need for renal replacement therapy is associated with high rates of morbidity and mortality. Hence, efforts to slow the progression of the disease are essential. Hypertension has been proven to be an independent risk factor for faster decline of glomerular filtration rate in renal patients, but studies involving only children with CAKUT are scarce. We performed a literature review to explore the association of hypertension with faster chronic kidney disease progression in children with CAKUT and also treatment options in this condition. A recent study reported an annual decline in GFR of 1.8 ml/min/1.73 m^2^ among hypertensive patients with non-glomerular CKD, compared with 0.8 ml/min/1.73 m^2^ in normotensive children. A multicenter prospective cohort in Brazil showed that a 1-unit increase in systolic blood pressure Z-score was associated with a 1.5-fold higher risk of disease progression. Since renin-angiotensin-aldosterone system activation is the most important mechanism of hypertension in these children, the first-line therapy involves the use of inhibitors of this axis, including angiotensin-converting enzyme inhibitors and angiotensin II receptor blockers type I, which also promote an anti-fibrotic effect. Recent studies have shown a good safety profile for use in patients with chronic kidney disease and also in those with solitary kidneys. Hypertension is an independent risk factor for kidney disease progression and should be promptly managed for renal protection, especially among patients with CAKUT, the primary cause of chronic kidney disease in the pediatric population.

## Introduction

Congenital anomalies of the kidney and urinary tract (CAKUT) are the primary cause of chronic kidney disease (CKD) in the pediatric population (1–4). Bilateral renal hypoplasia and dysplasia, with or without concomitant urinary tract malformation, are present in over 50% of children and adolescents requiring renal replacement therapy (2). According to data published for the Chronic Kidney Disease in Children (CKiD) cohort in 2015, of the 689 children involved, 76% had a non-glomerular cause for CKD, of which 69% were CAKUT-associated: 25% obstructive uropathy; 21% aplasia, hypoplasia or renal dysplasia; 19% reflux nephropathy; and 4% other CAKUTs (3). For previous registries reporting CKD etiology in infancy, the NAPRTCS found CAKUT in 48% of cases and the ItalKid in 58% (5, 6).

Many CAKUT patients will progress to end-stage renal disease (ESRD) because the congenital reduction in nephron mass ultimately overloads the remaining nephrons. In severe dysplasia cases, ESRD occurs in the first years of life, while in other malformations there is an initial transient period during which glomerular filtration rate (GFR) can increase, leading to hypertrophy of the remaining nephrons. This period can span several years and is generally followed by a phase of stability. Progressive loss of residual renal function occurs and often, at between 15 and 25 years of age, these patients require renal replacement therapy (2, 7–9). In a population-based registry of children with CAKUT (ItalKid Study), the risk of progressing to ESRD by the age of 20 was 68% (6).

ESRD is associated with high morbidity and mortality rates and therefore strategies to reduce the rate of CKD progression and thus delay renal replacement therapy can be crucial for improving life expectancy and quality of life of patients. Concerted efforts have been made in recent years to elucidate the risk factors associated with CKD progression and to provide treatment for renal protection. Hypertension has been shown to be one of these risks. Although studies involving only children with CAKUT are scarce, we performed a literature review to explore the association of hypertension with faster chronic kidney disease progression in children with CAKUT and also treatment options in this condition.

## Hypertension as a Risk Factor

A number of studies have shown that high blood pressure plays a role as an independent risk factor for faster GFR decline in renal patients (2, 3, 7, 10–13). In 1997, Wingen et al. confirmed the relationship of systolic blood pressure (SBP) with CKD progression, independently of proteinuria and protein intake (14). The trial was designed to test the effects of a low-protein vs. conventional diet on CKD progression during a 2–3 year period, while other factors such as BP were also monitored. The 284 patients registered at the 25 centers were aged 2–18 years and had CKD stage 3–4. On multivariate analysis, only hypertension (defined as systolic blood pressure >120 mmHg) and proteinuria (24-h urine protein >50 mg/kg) were independently associated with GFR decline. In a 2007 study, González Celedón et al. also found that hypertension contributed to more rapid renal function deterioration in children with CKD secondary to renal dysplasia and CAKUT (8).

In 2015, a study from the CKiD cohort showed that children aged 1–16 years with CKD stage 2–4 of non-glomerular origin (CAKUT and genetic diseases) had a mean annual GFR decline of 0.8 ml/min/1.73 m^2^ if normotensive and without proteinuria. In the presence of hypertension, however, this annual decrease in GFR rose to 1.8 ml/min/1.73 m^2^ (3).

A study conducted by our group in Brazil on data from a prospective multicenter cohort involving 209 children aged 1–17 years with CKD stages 3 and 4, found that 73% of the patients had CAKUT as the CKD etiology. A 31% greater risk of CKD progression was noted in patients with high BP at first visit in the study. A one unit increase in Z-score for systolic BP at start of follow-up was associated with a 1.3-fold higher risk of attaining the combined outcome of the study (death or need for renal replacement therapy or 50% decline in estimated GFR) (15).

Hypertension is a risk factor that generally develops early in pediatric patients with CKD and consequently has a high prevalence in this population. In reports of the North American Pediatric Renal Trials and Collaborative Studies (NAPRTCS) for 1994 and 2001, an estimated 67% of the 3,861 children with CKD were hypertensive (16). Hypertension was defined as systolic BP, diastolic BP or both, exceeding the 95th percentile, where measurements were taken during medical visits. In a report from the CKiD published in 2008, the prevalence of hypertension or high BP was 54% (17). Most surprisingly, in both of these analyses, one-third to a half of hypertension cases were either previously undiagnosed or uncontrolled.

The underlying mechanism of the BP effect on CKD progression is similar to that hypothesized to explain the BP effect on the cardiovascular system, with high BP playing a major role in causing vascular stiffening, secondarily impacting renal microcirculation (12). Many different pathophysiologic mechanisms result in hypertension, but renin-angiotensin-aldosterone system (RAAS) activation plays an important role. Areas of renal hypoperfusion such as cysts, scarring, dysplastic tissue, and endothelial injury, produce over-secretion of renin and consequent increase in Angiotensin II and Aldosterone (18, 19). Angiotensin II is a potent vasoconstrictor, besides promoting sympathetic activation. It also induces proliferation of smooth muscle cells and increases glomerular and tubular expression of several growth factors, cytokines and chemokines, which can result in glomerular hypertrophy, sclerosis, tubulointerstitial inflammation, and fibrosis (7, 9, 20, 21). Angiotensin II is now recognized as a proinflammatory agent able to modulate immunologic and inflammatory responses in endothelial, renal tubular and smooth muscle cells, such as chemotaxis, proliferation and differentiation of monocytes into macrophages (22), whereas aldosterone, primarily promotes salt retention. Of equal importance, endothelium activation by high BP is followed by endothelial dysfunction, which results in endothelial disintegration if the aggressive stimulus persists. Over time, vascular rarefaction occurs, which can result in reduced tissue perfusion and consequent hypoxia. Vascular rarefaction in capillaries of the renal medulla is central to hypoxia and tissue damage secondary to endothelial damage (22).

However, it is noteworthy that pediatric CAKUT patients are generally at lower risk of developing hypertension than patients with glomerulopathies. This is probably because children with CAKUT often have tubular dysfunction associated with vasopressin insensitivity, with consequent salt and water loss (18).

## High Blood Pressure Treatment

Controlling BP, therefore, becomes fundamental in the management of CKD patients to both reduce traditional cardiovascular risk and preserve residual renal function over the long-term. Life-style changes with dietary modification and physical activity are essential in this control, supporting drug therapy. Some studies in adult CKD patients have shown that dietary salt restriction can help better control BP, and also favor the anti-hypertensive and anti-proteinuric effect of RAAS blockers (23). All cases of secondary renal hypertension, however, require drug therapy. The Kidney Disease Improving Global Outcomes (KDIGO) guideline in 2012 is the only one that explicitly establishes a cut-off point for starting use of the drugs, at a BP >90th percentile for children with CKD (24). Among pharmacological measures, the first-line drugs for treating hypertension in CKD are RAAS inhibitors, including angiotensin-converting enzyme (ACE) inhibitors and angiotensin II receptor blockers type I (ARB), because they act on the primary mechanism causing hypertension and promote proteinuria reduction, also important for delaying progression of CKD (7, 11, 20, 25, 26). This approach is supported by evidence from the CKiD, showing that children in use of RAAS blockers have better BP control than patients using other classes of anti-hypertensives (17). In 2012, Samuels et al. also showed that patients using ACE inhibitors (ACEI) were 89% more likely to have a normal blood pressure on ambulatory blood pressure monitoring (ABPM) than those who did not report using ACEI (27).

The use of more than one class of antihypertensive is sometimes required to achieve sufficient BP control. Dual therapy combining ACEI and ARB is, in contrary to adults, not precluded in children. It is not indicated to adults, given there is no evidence supporting its use, in addition to data from studies that raise safety concerns (24, 28). However, to children, this combination has demonstrated an additive anti-hypertensive and anti-proteinuric effect in comparison to the maximal dose of ACEIs (29). At this time, more studies are necessary to support routine use of ACEI/ARB therapy in childhood.

Another promising class is beta-blockers, which have the added effect of reduction of renin and proteinuria. Diuretics can be useful for hypervolemia situations. Calcium channel blockers are not recommended as monotherapy, since they fail to reduce proteinuria, although may be useful as an additional agent (28).

Many anti-hypertensive agents currently used in children are administered off-label. Historically, explicit approval for pediatric use was generally not applied by manufacturers because clinical trials in children are more difficult than trials in adult patients for ethical, biometric, and practical reasons (20). This is exacerbated in children with CKD, who are often excluded from clinical studies. Consequently, safety and efficacy data for these drugs are lacking in this population. In an effort to clarify these doubts, in 2018 Watt et al. compiled available data (30), assessing 10 drugs submitted to the US Food and Drug Administration (FDA) for pediatric labeling from 1998 to 2005 (amlodipine, benazepril, enalapril, felodipine, fosinopril, irbesartan, lisinopril, losartan, quinapril, and ramipril). Each submission included a multicenter placebo-controlled trial testing safety and efficacy. Surprisingly, no significant difference was observed in the incidence of adverse events in children with decreased renal function (estimated GFR <90 ml/min/1.73 m^2^) compared with those with normal renal function (eGFR ≥ 90 ml/min/1.73 m^2^). The authors concluded that the inclusion of these drugs for the treatment of this patient group should be considered. However, it is important to note that all of the trials excluded children with severe renal dysfunction (eGFR < 30 ml/min/1.73 m^2^).

Nevertheless, there is some reticence in prescribing RAAS blockers to CKD patients. These concerns are grounded on the potential acute decline of GFR, hyperkalemia, and worsening of the anemia associated with the drug. Wuhl et al. analyzed these adverse effects using data from the ESCAPE trial, a prospective, randomized controlled trial performed in Europe involving children aged 3–18 years with CKD stages 2–4. The primary objective of the study was to assess the effect of strict control of blood pressure and of ACEI in CKD progression in pediatric patients. The agent used was ramipril and it was discontinued due to adverse effects in only 2.4% of the patients. Serum creatinine increased in 1.3% (5 out of 352 patients) during the first 6 months of treatment, confirmed by the decline in calculated creatinine clearance of over 25%. The incidence of this event did not differ from the pretreatment observation period. In addition, ramipril had to be discontinued due to persistent hyperkalemia in only one patient (0.3%), vs. 1.2–1.6% in adult trials, and mean serum potassium increased by only 0.3 mmol/L. With regard to anemia, it is acknowledged that RAASs can alter hematopoiesis and in the study, there was a drop in mean hemoglobin levels of 0.6 g/dL in 2 months, and stabilization thereafter. Thus, the safety profile of the drug in children appears to be good (20).

Another controversial issue is the use of RAAS inhibitors in patients with congenital or acquired solitary kidneys. According to literature reports, RAAS plays a central role in maintaining renal function in patients with a single kidney, being essential for the compensation mechanism. However, its degree of expression can influence progression of renal injury. Recently, Simeoni et al. carried out a literature review assessing the effect of the drug in these patients (9). Despite the scarcity of related data, it was suggested that anti-RAAS agents also have a renoprotective effect in patients with a single kidney, with little evidence of serious adverse effects. In small children, the exception is that the use of ARB would be more appropriate than ACEI, given that angiotensin II levels are critical in supporting the development and full maturation of the kidneys, and should not be completely inhibited.

## Blood Pressure Target

In 2009, the above-mentioned ESCAPE trial addressed not only choice of therapy but also target BP (11). The objective was to assess the effect of strict blood-pressure control (below 50th percentile) and progression of renal failure in children by comparing against conventional BP control (50–90th percentile). All patients in the study were administered the same fixed dose of ramipril. Additional reduction in the intensified blood-pressure control group, when necessary, was done by introducing antihypertensive agents that were not RAAS antagonists. The study found a 35% lower risk of either 50% loss of renal function or progression to ESRD in 5 years when BP control was intensified (BP below 50th percentile for age) compared to the conventional target (BP between 50 and 90th percentile). This effect was present in patients with glomerulopathy, dysplasia, and/or renal hypoplasia, not encompassing other congenital or hereditary nephropathies. Partly on the strength of these findings, the 2016 guidelines of the European Society of Hypertension (ESH) endorsed that the target BP in children with CKD should be below the 50th percentile in those with proteinuria and below the 75th in patients without proteinuria (31). This recommendation contradicts the previous guidance of the 4th Task Force Report and of the Kidney Disease Outcomes Quality Initiative (K/DOQI), both of 2004, which indicate treatment for BP values below the 90th percentile, based on data extrapolated from adult patient reports (28, 32, 33).

The most recent guidelines were published by the Kidney Disease Improving Global Outcomes (KDIGO) in 2012 and by the American Academy of Pediatrics (AAP)—Clinical Practice Guideline for Screening and Management of High Blood Pressure in Children and Adolescents (CPG) in 2017 (24, 25). In both cases, the therapy target is also stricter and the documents suggest that BP should be reduced to below the 50th percentile, unless there are hypotension symptoms. The KDIGO stipulates control based on casual BPs while the AAP adopts ABPM.

## How to Monitor Blood Pressure?

The current American Heart Association guidelines consider ABPM the gold standard for monitoring BP control (34). The justification for this guideline is based on the stronger association between ambulatory BP measurements and lesions in target organs, compared with BP readings taken in the office, observed in children with and without CKD in multiple observational studies (35–37). In addition, ABPM is highly useful for diagnosing masked hypertension, nocturnal hypertension, and abnormal BP loads, not possible using casual BPs in the office (26, 38). In their 2017 guideline, the AAP advises that, irrespective of apparent control of BP based on office measurements, children and adolescents with CKD and history of hypertension should have BP assessed by ABPM at least yearly to screen for masked hypertension (25). In a 2012 report for the CKiD cohort, 332 children with CKD underwent ABPM one year after study entry and 35% were subsequently diagnosed with masked hypertension, defined as a normal casual BP yet abnormal ABPM reading (27). The report provided further support for the AAP recommendation of performing annual ABPM.

However, a more recent study published in 2018 analyzing 513 children from the CKiD cohort, concluded that systolic BPs carefully collected using the standardized protocol of auscultatory technique, were not inferior to the ambulatory BPs for stratifying risk in children with CKD. Both approaches can yield similar prognostic information, including on the risk of progression to ESRD (38). This represents important information, particularly for facilities in which ABPM is not feasible or for children in which this type of monitoring is not possible.

## Proteinuria and Hypertension

Proteinuria, besides hypertension, is another important independent risk factor for the progression of renal disease (3, 8, 21). However, they are in some ways inter-related. Large studies in adults have shown that blood pressure control alone has an anti-proteinuric effect (39, 40). This can be explained by the fact that the blood pressure increase leads to elevated intraglomerular pressure, hyperfiltration and increased urine excretion of proteins. Associated with this, the main pathophysiologic mechanism involved in hypertension in CKD is RAAS activation with increase in angiotensin II. This also promotes local increase in intraglomerular pressure, besides stimulating the local release of cytokines and activating inflammatory pathways, aggravating glomerular hypertrophy, sclerosis, tubulointerstitial inflammation and fibrosis, causing further renal damage and hence proteinuria. Therefore, hypertension control should be evaluated in conjunction with proteinuria, and the choice of agents that inhibit RAAS becomes irrefutable since they provide superior renoprotection.

## Limitations

Much of the data resumed in the present manuscript stems from studies and guidelines aimed at CKD children and not specifically CAKUT children since there is limited literature for this. However, CAKUT is the leading cause of CKD in childhood, and we suppose that data from CKD patients represent mainly children with CAKUT.

## Conclusion

Congenital anomalies of the kidney and urinary tract (CAKUT) are the primary cause of CKD in children and these patients will often progress to ESRD and require renal replacement therapy. A number of risk factors associated with kidney disease progression were assessed and hypertension proved deleterious in most of the studies.

In summary, the available evidence suggests that management of BP is essential in children with CAKUT-associated CKD in order to slow the progression of the disease. RAAS inhibitors are the first-line medications for the efficacy of their anti-hypertensive, anti-proteinuric, and potential renal anti-fibrotic effects. Target ABPM values should be below the 50th percentile in proteinuric and below the 75th percentile in non-proteinuric CKD children.

## Author Contributions

PK: conceived the manuscript and revised the whole text. MG: conceived, wrote, and revised the manuscript.

### Conflict of Interest Statement

The authors declare that the research was conducted in the absence of any commercial or financial relationships that could be construed as a potential conflict of interest.
